# Approved therapies in the IgA nephropathy armamentarium: a summary of the evidence

**DOI:** 10.1016/j.kisu.2026.03.001

**Published:** 2026-07-20

**Authors:** Sayna Norouzi, Richard A. Lafayette

**Affiliations:** 1 Division of Nephrology, Loma Linda University Medical Center, Loma Linda, California, USA; 2 Division of Nephrology, Department of Medicine, Stanford University School of Medicine, Stanford University, Stanford, California, USA

**Keywords:** atrasentan, IgA nephropathy, iptacopan, Nefecon, sibeprenlimab, sparsentan

## Abstract

Historically, IgA nephropathy (IgAN) was managed using supportive care, with a suggested 6 months of immunosuppressive therapy considered for patients at high risk of progressive kidney function decline (proteinuria, >0.75–1 g/d) despite ≥90 days of optimized supportive care. The evolving treatment landscape includes agents that target immunologic aspects of IgAN or better preserve kidney function, or both. Recent approvals include Nefecon, sparsentan, iptacopan, atrasentan, and sibeprenlimab. Nefecon, a targeted-release formulation of budesonide, is the only US Food and Drug Administration and European Medicines Agency fully approved agent that is designed to target the primary source of IgAN: galactose-deficient IgA1 production in the gut mucosa. Phase 3 data showed that Nefecon slowed estimated glomerular filtration rate decline and decreased proteinuria versus placebo, with an acceptable safety profile. Sparsentan, a fully US Food and Drug Administration– and European Medicines Agency–approved dual endothelin A/angiotensin II receptor antagonist, affects the generic responses to IgAN-induced nephron loss, potentially including glomerular inflammation and fibrosis. Sparsentan demonstrated a significant reduction in proteinuria and estimated glomerular filtration rate decline compared with irbesartan. Iptacopan inhibits complement factor B and has received accelerated US Food and Drug Administration approval. Interim phase 3 study data have shown that iptacopan significantly reduces proteinuria when compared with placebo. The endothelin A receptor antagonist atrasentan and A proliferation binding ligand inhibitor sibeprenlimab have also received accelerated US Food and Drug Administration approval, based on interim phase 3 results showing significant proteinuria reduction versus placebo in patients with IgAN. This expanded clinical armamentarium offers clinicians and patients greater choice and improved disease control in IgAN management.

Clinical research demonstrating the benefits of therapeutic intervention for kidney diseases has often been challenging, because long follow-up times are required for “hard” clinical end points, such as mortality or end-stage kidney disease. In 2016, the National Kidney Foundation collaborated with the US Food and Drug Administration and the European Medicines Agency and identified the estimated glomerular filtration rate (eGFR) slope and reduction in proteinuria as likely surrogate end points that could predict long-term treatment benefit on kidney outcomes in patients with IgA nephropathy (IgAN).^1^, ^2^, ^3^ These have now become somewhat standard outcomes for evaluating new therapeutic candidates for accelerated approval for the treatment of IgAN, with a subsequent definitive approval if the drug is proven effective for moderate-term eGFR preservation over the course of a trial.^3^, ^4^, ^5^ This topic is discussed in more detail elsewhere in this supplement, in the article titled “Management of IgA nephropathy and the expanding role of immunomodulation” by Canetta and Reich.^6^

**Key Learning Points**

• The treatment landscape of IgA nephropathy (IgAN) is rapidly evolving to include a growing number of therapies that target the immunologic aspects of the disease, as well as the consequences of IgAN-induced nephron loss.
• Given the recent approvals of new treatments, the clinical armamentarium for managing IgAN has already expanded greatly, providing clinicians and patients alike with greater choice.

Until recently, treatment options for patients with IgAN have been limited. The mainstay of therapy has been supportive care, and a suggestion to consider 6 months of immunosuppressive therapy (usually systemic glucocorticoids) in patients at continued high risk of progressive kidney function decline (proteinuria, >0.75–1 g/d) despite ≥90 days of supportive care. The updated 2025 Kidney Disease: Improving Global Outcomes (KDIGO) guidelines “recommend that all patients with IgAN”^7^^(page S46)^ be treated with an optimized, maximally tolerated dose of a renin-angiotensin system (RAS) inhibitor to manage the generic response to IgAN-induced nephron loss. However, sparsentan, a dual endothelin A (ETA) and angiotensin II receptor antagonist (DEARA), is suggested instead of an RAS inhibitor for “patients who are at risk of progressive loss of kidney function”^7^^(page S47)^ (proteinuria, ≥0.5 g/d or equivalent).^7^ This suggestion is based on the findings of the PROTECT trial.^7^^,^^8^ The 2025 KDIGO guidelines also suggest that patients at risk of progressive loss of kidney function receive a sodium-glucose cotransporter-2 inhibitor (SGLT2i) in addition to RAS inhibitor or DEARA.^7^ This recommendation was based on the results of the analysis of patients with IgAN in the Dapagliflozin and Prevention of Adverse Outcomes in Chronic Kidney Disease (DAPA-CKD) trial, which demonstrated that use of an SGLT2i had beneficial effects on eGFR and urine albumin-creatinine ratio.^7^^,^^9^ The 2025 KDIGO guidelines also suggest Nefecon treatment for patients at risk of progressive loss of kidney function to manage IgAN-specific drivers of nephron loss. This is based on the results of the NefIgArd trial.^7^^,^^10^ Both the PROTECT and Nefecon in Patients with Primary IgA Nephropathy at Risk of Progressing to End-Stage Renal Disease (NefIgArd) trials are discussed in more detail later in this article. Given the recognition of surrogate end points in IgAN, as described above, new therapies have been approved, and many others are being evaluated in clinical trials, including therapies that target IgAN-specific drivers of disease and nephron loss.

This review discusses recently approved treatment options for the management of IgAN and provides a detailed overview of the efficacy and safety data that led to the approval of these therapies. These are summarized in Table 1 (Nawaz N, Thomas RC, Barratt J. Impact of nefecon on complement pathways in IgA nephropathy: an analysis of lectin, alternative, and terminal pathways [abstract]. Presented at: American Society of Nephrology Kidney Week. October 23–27, 2024; San Diego, CA. Abstract FR-PO859; and Thomas RC, Nawaz N, Barratt J. Specificity of nefecon in targeting pathogenic IgA in IgA nephropathy while preserving systemic humoral immunity [abstract]. Presented at: American Society of Nephrology Kidney Week. October 23–27, 2024; San Diego, CA. Abstract FR-PO894).^8^^,^^10^, ^11^, ^12^, ^13^, ^14^, ^15^, ^16^, ^17^, ^18^, ^19^, ^20^, ^21^, ^22^, ^23^, ^24^, ^25^, ^26^, ^27^, ^28^, ^29^, ^30^, ^31^, ^32^, ^33^, ^34^, ^35^, ^36^, ^37^, ^38^, ^39^, ^40^, ^41^, ^42^, ^43^, ^44^, ^45^, ^46^

**Table 1:** Summary of phase 3 randomized clinical trials

Category	Nefecon	Sparsentan	Iptacopan^a^	Atrasentan^a^	Sibeprenlimab^a^
Mode of action	Targeted-release budesonide	Dual ETA and AngII receptor antagonist	Factor B inhibitor	ETA receptor antagonist	APRIL inhibitor
IgAN approval(s)					
FDA	Accelerated/full: December 2021/December 2023^11^^,^^12^	Accelerated/full: February 2023/September 2024^13^^,^^14^	Accelerated: August 2024^15^	Accelerated: April 2025^16^	Accelerated: November 2025^17^
EMA	Conditional/full: July 2022/July 2024^18^^,^^19^	Conditional/full: April 2024/April 2025^20^^,^^21^	–	–	–
Other	NMPA in China full approval: 2023^22^ PABM in Macau full approval: 2023^23^ MFDS in South Korea full approval: 2024^24^ TFDA in Taiwan full approval: 2024^25^ HKDH in Hong Kong full approval: 2024^26^ HSA in Singapore full approval: 2024^27^	Swissmedic temporary authorization: October 2024^28^	–	–	–
Indication(s)					
FDA	Reduce the loss of kidney function in adults with primary IgAN who are at risk for disease progression^29^	Slow the loss of kidney function in adults with primary IgAN who are at risk for disease progression^30^	Reduction of proteinuria in adults with primary IgAN at risk of rapid disease progression, generally a UPCR ≥1.5 g/g^31^	Reduction of proteinuria in adults with primary IgAN at risk of rapid disease progression (generally, UPCR ≥1.5 g/g)^32^	Reduction of proteinuria in adults with primary IgAN at risk of rapid disease progression^17^
EMA	Treatment of adults with primary IgAN with a urine protein excretion ≥1.0 g/d (or UPCR ≥0.8 g/g)^33^	Treatment of adults with primary IgAN with a urine protein excretion ≥1.0 g/d (or UPCR ≥0.75 g/g)^34^	–	–	–
Administration route and dosing frequency	16 mg orally once daily for 9 mo^29^^,^^33^	200 mg orally once daily, increased after 14 d to 400 mg once daily, as tolerated^30^^,^^34^	200 mg orally twice daily^31^	0.75 mg orally once daily^32^	400 mg injected s.c. once every 4 wk^17^
Pivotal clinical trial	NefIgArd (NCT03643965)^10^^,^^35^	PROTECT (NCT03762850)^8^^,^^36^	APPLAUSE-IgAN (NCT04578834)^37^^,^^38^	ALIGN (NCT04573478)^39^^,^^40^	VISIONARY (NCT05248646)^41^^,^^42^
Study design	Randomized, placebo-controlled, double-blind, phase 3^10^	Randomized, active-controlled, double-blind, phase 3^8^	Randomized, placebo-controlled, double-blind, phase 3^37^	Randomized, placebo-controlled, double-blind, phase 3^39^	Randomized, placebo-controlled, double-blind, phase 3^41^
Patients and dosing	≥18 y; UPCR ≥0.8 g/g or proteinuria ≥1 g/d; eGFR 35–90 ml/min per 1.73 m^2^; Nefecon 16 mg/d^b^ (n = 182); placebo^b^ (n = 182); median time from biopsy to study entry: 2.5 y (IQR, 0.6–6.8 y)^10^	≥18 y; proteinuria ≥1 g/d; eGFR ≥30 ml/min per 1.73 m^2^; BP ≤150/100 mm Hg; Sparsentan 400 mg once daily (n = 202); irbesartan 300 mg once daily (n = 202); median (IQR) time from biopsy to informed consent: 4.0 (1.0–10.0) y^8^	≥18 y; UPCR ≥1 g/g; eGFR ≥30 ml/min per 1.73 m^2^; Iptacopan 200 mg twice daily^b^ (n = 125); placebo^b^ (n = 125); median (IQR) time from biopsy in the iptacopan group: 1.3 (0.5–2.8) y^37^	≥18 y; total urine protein ≥1 g/d; eGFR ≥30 ml/min per 1.73 m^2^; Atrasentan 0.75 mg once daily (n = 135); placebo (n = 135); mean duration of disease: 5.6 y^39^	≥18 y; UPCR ≥0.75 g/g or proteinuria ≥1 g/d; eGFR ≥30 ml/min per 1.73 m^2^; Sibeprenlimab 400 mg Q4W (n = 152), 26 total doses; placebo (n = 168); median time from initial biopsy to randomization (range) in the sibeprenlimab group: 1.3 (0.1–23.7)^41^
Primary end point	**Time-weighted average of eGFR over 2 y** 5.05 ml/min per 1.73 m^2^ benefit in favor of Nefecon vs. placebo (*P* < 0.0001); Time-weighted average change of Nefecon –2.47 vs. placebo –7.52 ml/min per 1.73 m^2^^10^	**Change from baseline in 24-h UPCR at 36 wk** Greater reduction in UPCR with sparsentan: −50% vs. Irbesartan −15% (*P* < 0.0001)^43^	**Change from baseline in 24-h UPCR at month 9** Mean UPCR was 38% lower with iptacopan than with placebo (*P* < 0.001)^44^	**Change from baseline in 24-h UPCR at 36 wk** Mean geometric UPCR was 36% (*P* < 0.001) lower with atrasentan than with placebo^39^	**Change from baseline in 24-h UPCR at month 9** Geometric LS mean UPCR 24-h was 51.2% lower with sibeprenlimab than placebo (*P* < 0.001)^41^
Key secondary end point(s)	**Composite end point of time to confirmed 30% reduction in eGFR or kidney failure** Significantly delayed with Nefecon vs. placebo (HR, 0.45; *P* = 0.0014)^10^ **Reduction in proteinuria** A significant reduction was observed with Nefecon vs. placebo, with a 41% reduction in time-averaged UPCR between 12 and 24 mo (*P* < 0.0001)^10^	**Chronic 2-y slope of the eGFR (weeks 6–110)** Sparsentan −2.7 vs. irbesartan −3.8 ml/min per 1.73 m^2^ per year (difference, 1.1 ml/min per 1.73 m^2^ per year; *P* = 0.037)^8^	eGFR data not currently available	**Change from baseline in eGFR (CKD-EPI)** Secondary end points will be tested at the final analysis^39^	eGFR data currently not available^41^
Most commonly reported TEAEs	Nefecon vs. placebo: Peripheral edema: 31 (17%) vs. 7 (4%); Hypertension: 22 (12%) vs. 6 (3%); Muscle spasms: 22 (12%) vs. 7 (4%); Acne: 20 (11%) vs. 2 (1%); and Headache: 19 (10%) vs. 14 (8%)^10^	Sparsentan vs. irbesartan: COVID-19: 53 (26%) vs. 46 (23%); Hyperkalemia: 32 (16%) vs. 26 (13%); Peripheral edema: 31 (15%) vs. 24 (12%); Dizziness: 30 (15%) vs. 13 (6%); Headache: 27 (13%) vs. 26 (13%); Hypotension: 26 (13%) vs. 8 (4%); and Hypertension: 22 (11%) vs. 28 (14%)^8^	Iptacopan vs. placebo: COVID-19: 31 (14%) vs. 37 (17%); Upper respiratory tract infection: 20 (9%) vs. 16 (7%); Nasopharyngitis: 11 (5%) vs. 16 (7%); Headache: 9 (4%) vs. 12 (5%); and Hypertension: 4 (2%) vs. 13 (6%)^44^	Atrasentan vs. placebo: COVID-19: 35 (21%) vs. 37 (22%); Nasopharyngitis: 17 (10%) vs. 10 (6%); Peripheral edema: 15 (9%) vs. 11 (7%); Anemia: 11 (7%) vs. 2 (1%); Pyrexia: 11 (7%) vs. 7 (4%); and Upper respiratory tract infection: 11 (7%) vs. 9 (5%)^39^	Sibeprenlimab vs. placebo: Upper respiratory tract infections: 38 (14.7%) vs. 35 (13.9%); Injection-site erythema: 34 (13.1%) vs. 30 (12.0%); Injection-site pain: 26 (10.0%) vs. 23 (9.2%); Nasopharyngitis: 32 (12.4%) vs. 25 (10.0%); COVID-19: 25 (9.7%) vs. 17 (6.8%); Influenza: 21 (8.1%) vs. 16 (6.4%)^41^
Biomarker data	Significant reductions in Gd-IgA1, anti–IgA-IgG autoantibodies, immune complexes, and complement factor B vs. placebo at 9 mo; no significant difference in levels of total IgA or IgG, or antitetanus toxoid Igs vs. placebo^45^^c^	–	Reductions in complement biomarker levels, including plasma Bb, serum Wieslab, and plasma and urinary C5b-9, with iptacopan vs. placebo^44^^,^^46^	–	Reductions in Gd-IgA1, APRIL, IgA, IgG, and IgM with sibeprenlimab vs. minimal change with placebo^41^

–, data have not been published; AngII, angiotensin II; APRIL, A proliferation binding ligand; BP, blood pressure; C5b-9, complement 5b-9 or membrane attack complex; CKD-EPI, Chronic Kidney Disease Epidemiology Collaboration; COVID-19, coronavirus disease 2019; eGFR, estimated glomerular filtration rate; EMA, European Medicines Agency; ETA, endothelin A; FDA, US Food and Drug Administration; Gd-IgA1, galactose-deficient IgA1; HKDH, Hong Kong Department of Health; HR, hazard ratio; HSA, Health Sciences Authority; IgAN, IgA nephropathy; IQR, interquartile range; LS, least squares; MFDS, Ministry of Food and Drug Safety; NeflgArd, Nefecon in Patients with Primary IgA Nephropathy at Risk of Progressing to End-Stage Renal Disease; NMPA, National Medical Products Administration; PABM, Pharmaceutical Administration Bureau of Macau; Q4W, every 4 weeks; TEAE, treatment-emergent adverse event; TFDA, Taiwan Food and Drug Administration; UPCR, urine protein-creatinine ratio.

a This indication is approved under accelerated approval based on reduction of proteinuria. It has not been established whether this agent slows kidney function decline in patients with IgAN. Continued approval for this indication may be contingent on verification and description of clinical benefit in a confirmatory clinical trial.

b Plus optimized supportive care.

c Nawaz N, Thomas RC, Barratt J. Impact of nefecon on complement pathways in IgA nephropathy: an analysis of lectin, alternative, and terminal pathways [abstract]. Presented at: American Society of Nephrology Kidney Week. October 23–27, 2024; San Diego, CA. Abstract FR-PO859; and Thomas RC, Nawaz N, Barratt J. Specificity of nefecon in targeting pathogenic IgA in IgA nephropathy while preserving systemic humoral immunity [abstract]. Presented at: American Society of Nephrology Kidney Week. October 23–27, 2024; San Diego, CA. Abstract FR-PO894. Endpoints are presented in bold.

## Nefecon

Nefecon is an oral, targeted-release capsule formulation of budesonide approved in many countries for the treatment of IgAN.^10^^,^^29^ It was the first approved therapy specifically indicated for the treatment of IgAN,^12^^,^^18^ and is designed to be released in the distal ileum for maximal exposure of budesonide at the Peyer patches.^10^ It is the only currently approved agent that specifically targets the primary source of IgAN: the production of galactose-deficient IgA1 (Gd-IgA1) in the gut mucosa.^7^^,^^29^^,^^33^ By reducing Gd-IgA1 production, Nefecon affects “hit 1” of the 4-hit IgAN pathogenesis pathway and addresses the elevated levels of Gd-IgA1 seen in patients with IgAN; in doing so, it has a positive downstream effect on the other 3 hits in the pathway.^29^^,^^33^^,^^47^ Full approvals for Nefecon were based on the complete 2-year phase 3 NefIgArd clinical trial data (Table 1).^10^^,^^12^^,^^19^

### NefIgArd phase 3 trial

#### Trial design

**Figure 1:**
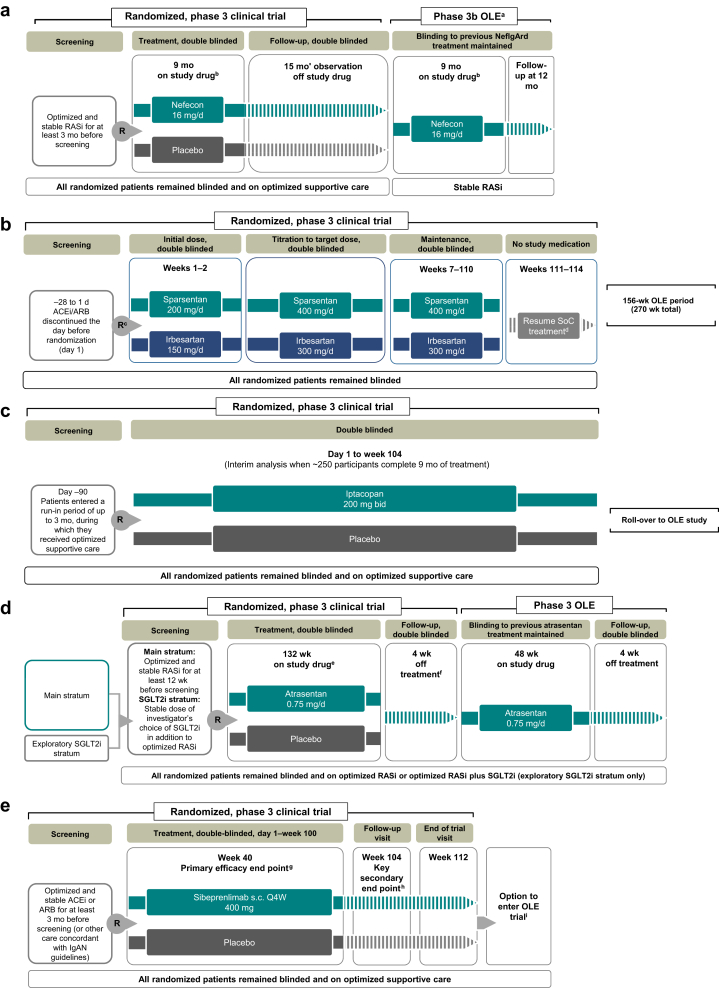
**Phase 3 clinical trials evaluating treatments for IgAN.** (**a**) NefIgArd phase 3 clinical trial design and the phase 3b open-label extension (OLE) study. (**b**) PROTECT phase 3 clinical trial design. (**c**) APPLAUSE-IgAN phase 3 clinical trial design. (**d**) ALIGN phase 3 clinical trial design and the phase 3 OLE. (**e**) VISIONARY phase 3 clinical trial design. ^a^The OLE enrolled patients with persistent proteinuria ≥1 g/d or urine protein-creatinine ratio (UPCR) ≥0.8 g/g and estimated glomerular filtration rate (eGFR) ≥30 ml/min per 1.73 m^2^ despite optimized renin-angiotensin system inhibition. Patients who completed a full 9-month (mo) course of Nefecon, 16 mg/d, without dose reductions were included. ^b^Followed by a 2-week (wk) taper at 8 mg/d. ^c^On day 1, patients were randomized 1:1 to sparsentan or irbesartan, stratified by eGFR value (30–<60 and ≥60 ml/min per 1.73 m^2^) and urine protein excretion (≤1.75 and >1.75 g/d). ^d^Following the blinded treatment period, treatment with study medication was discontinued. At this time, standard of care (SoC) treatment could be resumed, including treatment with RASi. The investigator could make additional adjustments in antihypertensive medications as clinically indicated to adequately control the patient’s blood pressure. ^e^Interim analysis was conducted at week 36. ^f^Final analysis was conducted at week 136. ^g^The 24-hour UPCR at 9 months compared with baseline. ^h^Annualized eGFR slope estimated over 24 months. ^i^Patients who completed ≥20 doses and the end-of-trial visit at week 112 were eligible to enter an OLE trial. ACEi, angiotensin-converting enzyme inhibitor; ARB, angiotensin receptor blocker; bid, twice daily; IgAN, IgA nephropathy; Q4W, every 4 weeks; R, randomization; RASi, renin-angiotensin system inhibitor; SGLT2i, sodium-glucose cotransporter-2 inhibitor.

NefIgArd (ClinicalTrials.gov Identifier: NCT03643965)^35^ was an international, multicenter, randomized, double-blind, placebo-controlled trial conducted in 132 hospital-based clinical sites across 20 countries in Europe, North and South America, and Asia Pacific (Figure 1a).^10^ After an initial 15- to 35-day screening period, 364 adult patients with IgAN were randomized 1:1 to receive treatment with Nefecon, 16 mg/d (n = 182), or matching placebo (n = 182) for 9 months, plus optimized supportive care (i.e., blood pressure control [target, <125/75 mm Hg], dietary sodium restriction, and stable dose of maximally tolerated or allowed RAS inhibition [angiotensin-converting enzyme inhibitor or angiotensin II type 1 receptor blocker] for the 3 months before randomization).^10^ Following a tapering period of 2 weeks, a further 15-month observational follow-up period was conducted, during which no study drug was administered but optimized supportive care was continued.^10^

Adult patients with biopsy-confirmed primary IgAN, persistent proteinuria (urine protein-creatinine ratio [UPCR], ≥0.8 g/g or proteinuria, ≥1 g/d) despite optimized supportive care for at least 3 months before randomization, and an eGFR of ≥35 to ≤90 ml/min per 1.73 m^2^ were eligible to enroll in the NefIgArd trial.^9^ Randomization was stratified according to baseline proteinuria (<2 or ≥2 g/d), baseline eGFR (<60 or ≥60 ml/min per 1.73 m^2^), and geographic region.^10^^,^^48^

#### Efficacy outcomes

Nefecon, 16 mg/d, for 9 months provided a statistically significant and clinically relevant reduction in eGFR decline and a durable reduction in proteinuria versus placebo.^7^^,^^10^^,^^48^ The primary end point for the full 2-year trial, 2-year time-weighted average of eGFR, showed a statistically significant 5.05 ml/min per 1.73 m^2^ eGFR treatment benefit in favor of Nefecon, 16 mg/d, versus placebo (*P* < 0.0001), with a time-weighted average change of –2.47 versus –7.52 ml/min per 1.73 m^2^ reported in the Nefecon and placebo arms, respectively.^10^

The eGFR benefit accrued after 9 months of treatment was maintained throughout the 15-month observational follow-up period, and at 24 months, there was a 6.11 ml/min per 1.73 m^2^ decline in eGFR in the Nefecon arm compared with a 12.0 ml/min per 1.73 m^2^ decline in the placebo arm.^10^ This corresponds to a difference in the 2-year eGFR total slope of 2.95 ml/min per 1.73 m^2^ per year (*P* < 0.0001) and represents 50% less deterioration of kidney function in Nefecon-treated patients compared with placebo recipients over the 2-year period.^10^ Time to 30% eGFR reduction from baseline or kidney failure was also significantly delayed with Nefecon versus placebo (hazard ratio, 0.45; *P* = 0.0014), with 21 (12%) and 39 (21%) patients having a confirmed event in the Nefecon and placebo arms, respectively.^10^

Reductions in proteinuria were also observed during this trial, with a significant 41% reduction in the time-averaged UPCR (measured over 12–24 months from baseline) with Nefecon versus placebo (*P* < 0.0001) and a 40% decrease in UPCR from baseline in Nefecon-treated patients compared with a 1% increase in patients receiving placebo.^9^ Furthermore, the percentage reduction (30% in Nefecon-treated patients) seen in UPCR at 24 months was similar to that achieved at the end of the 9-month treatment period in the Nefecon versus placebo arm.^10^

The clinical benefits seen with Nefecon were further supported by the significantly higher proportion of patients without microhematuria reported during the observation follow-up period in the Nefecon-treated arm versus the placebo arm (59% vs. 39%, respectively; *P* = 0.0001).^10^

In an open-label extension study (NCT04541043),^49^ patients who completed the NefIgArd trial with persistent proteinuria, ≥1 g/d, or UPCR ≥0.8 g/g and eGFR ≥30 ml/min per 1.73 m^2^ despite optimized RAS inhibition were assessed for the effects of a second 9-month Nefecon treatment course (Figure 1a). Results following the additional 9 months of treatment with Nefecon demonstrated a similar active treatment benefit on proteinuria and kidney function decline to that seen in the phase 3 NefIgArd study, regardless of whether the patient previously received Nefecon or placebo (Lafayette R, Kristensen J, Jones R, et al. NefIgArd open-label extension: efficacy and safety of nefecon in patients with IgAN who completed the 2-year phase 3 trial [abstract]. Presented at: American Society of Nephrology Kidney Week. October 23–27, 2024; San Diego, CA. Abstract FR-OR56).

The phase 4 NefXtend trial (NCT06712407)^50^ is underway and aims to evaluate the efficacy and safety of extended treatment with Nefecon for an additional 15 months after 9 months of Nefecon, 16 mg/d, in patients with IgAN and residual proteinuria in real-world clinical practice (Lafayette R. Investigating extended nefecon treatment beyond 9 months in patients with IgAN: the phase 4 NefXtend trial [poster]. Presented at: American Society of Nephrology Kidney Week. November 5–9, 2025; Houston, TX. Poster INFO14-TH).

#### Safety outcomes

Nefecon, 16 mg/d, was well tolerated in the NefIgArd trial, with a safety profile expected of a targeted-release oral budesonide product.^10^ During the observational follow-up period, the incidences of treatment-emergent adverse events (TEAEs) and treatment-emergent serious adverse events (TESAEs) were similar in both treatment arms. The most commonly reported TEAEs during treatment with Nefecon versus placebo were peripheral edema (31 [17%] vs. 7 [4%]), hypertension (22 [12%] vs. 6 [3%]), muscle spasms (22 [12%] vs. 7 [4%]), acne (20 [11%] vs. 2 [1%]), and headache (19 [10%] vs. 14 [8%]). These were generally nonserious AEs, of mild severity, and were reversible during or after treatment. One death due to coronavirus infection was reported during Nefecon treatment, and another patient previously treated with Nefecon died from a cerebral hemorrhage 10.5 months after the last dose; neither death was considered treatment related.^10^ Of the patients assessed in the NefIgArd open-label extension study, no new safety signals were observed following an additional 9 months of treatment with Nefecon (Lafayette R, Kristensen J, Jones R, et al. NefIgArd open-label extension: efficacy and safety of nefecon in patients with IgAN who completed the 2-year phase 3 trial [abstract]. Presented at: American Society of Nephrology Kidney Week. October 23–27, 2024; San Diego, CA. Abstract FR-OR56).

#### Predicting the long-term clinical benefit of Nefecon

A linear regression model was used to extrapolate the effect of Nefecon on the eGFR slope from the NefIgArd trial to predict the impact of Nefecon on the clinical outcome of kidney failure, eGFR <15 ml/min per 1.73 m^2^, or sustained doubling of serum creatinine in a real-world IgAN population.^10^^,^^51^ Modeling analyses were based on Leicester General Hospital registry data from patients with IgAN and matched to individual NefIgArd patients based on their UPCR and eGFR values.^51^ A median delay to the clinical outcome by 12.8 years (95% confidence interval [CI], 4.8–27.9 years) was predicted with Nefecon treatment, with a median time to outcome of 9.6 years for patients included in the registry receiving supportive care only versus 22.4 years for Nefecon-treated patients.

#### Biomarker data

Analyses of serum samples from the NefIgArd trial demonstrated that Nefecon treatment resulted in significant reductions in Gd-IgA1, anti–IgA-IgG autoantibodies, immune complexes, and complement factor B (a marker of the alternative complement pathway) at 9 months compared with placebo (Nawaz N, Thomas RC, Barratt J. Impact of nefecon on complement pathways in IgA nephropathy: an analysis of lectin, alternative, and terminal pathways [abstract]. Presented at: American Society of Nephrology Kidney Week. October 23–27, 2024; San Diego, CA. Abstract FR-PO859; and Thomas RC, Nawaz N, Barratt J. Specificity of nefecon in targeting pathogenic IgA in IgA nephropathy while preserving systemic humoral immunity [abstract]. Presented at: American Society of Nephrology Kidney Week. October 23–27, 2024; San Diego, CA. Abstract FR-PO894.*)*. By contrast, there was no significant difference in levels of total IgA or IgG, or antitetanus toxoid Igs compared with placebo, indicating that systemic humoral immunity was unaffected. These results are supported by similar earlier biomarker analysis findings from the phase 2b The Effect of Nefecon® in Patients With Primary IgA Nephropathy at Risk of Developing End-stage Renal Disease (NEFIGAN) trial, which highlighted a significant reduction in levels of both serum Gd-IgA1 and IgA/IgG immune complexes with Nefecon treatment, strongly supporting a disease-modifying action of Nefecon in IgAN.^45^ This topic is discussed further in the article in this supplement titled “The expanding role of biomarkers in the management of IgA nephropathy” by Jain and Rizk.^52^

### Key considerations when using Nefecon

Treatment with Nefecon does not require specific monitoring. However, as the active ingredient is budesonide, there is the potential for systemic effects that are general to glucocorticoid use, such as hypercorticism, adrenal axis suppression, immunosuppression, and increased risk of infection,^29^^,^^33^ which should be discussed with a patient before commencing Nefecon. Such effects are usually mild and reversible on treatment cessation.

## Sparsentan

Sparsentan is a DEARA working subsequent to the 4-hit pathway to manage the generic responses to IgAN-induced nephron loss, potentially including glomerular inflammation and fibrosis.^30^^,^^34^^,^^37^ ETA and angiotensin II interact with membrane receptors on the surface of most cell types intrinsic to the kidney and induce several hemodynamic and structural changes in glomeruli, potentially leading to proteinuria, progressive glomerulosclerosis, inflammation, and fibrosis.^53^ Sparsentan was granted full US Food and Drug Administration approval in 2024 and is indicated to slow kidney function decline in adults with primary IgAN who are at risk for disease progression (Table 1).

### PROTECT phase 3 trial

#### Trial design

PROTECT (NCT03762850),^36^ a double-blind, randomized, active-controlled, phase 3 international clinical trial, was conducted across 134 clinical sites in 18 countries throughout the Americas, Asia, and Europe.^8^ This trial was designed to evaluate the safety and efficacy of continuous treatment with sparsentan versus an active comparator (irbesartan) in adult patients with IgAN (Figure 1b). Adult patients with biopsy-confirmed primary IgAN and proteinuria ≥1 g/d, an eGFR ≥30 ml/min per 1.73 m^2^, and blood pressure ≤150/100 mm Hg, despite optimal RAS inhibition for 12 weeks, were randomized (1:1) to receive sparsentan (n = 202) at an initial dose of 200 mg once daily for 2 weeks, followed by a target dose of 400 mg once daily, or irbesartan (n = 202), 150 mg once daily, for 2 weeks, followed by a target dose of 300 mg once daily.^8^ The 114-week trial included up to 110 weeks of study treatment period, followed by 4 weeks without study treatment but with standard of care (RAS inhibition) permitted during this time.^8^ Patients could then be followed during a 156-week open-label extension period.^8^

#### Efficacy outcomes

Over the 110-week treatment period, sparsentan resulted in significant reductions in proteinuria and in preservation of kidney function versus irbesartan.^7^ In a prespecified interim analysis, sparsentan treatment resulted in a significant and clinically meaningful reduction in proteinuria at 36 weeks (primary end point) versus irbesartan (–50% vs. −15%), resulting in a between-group relative reduction of 41% (least-squares mean ratio, 0.59; 95% CI, 0.51−0.69; *P* < 0.0001).^43^ This reduction seen at 36 weeks with sparsentan was maintained throughout the study period.^8^

Prespecified key secondary end points included total eGFR slope (day 1–week 110) and chronic eGFR slope (weeks 6–110).^8^ Total slope reflects the entire treatment period, including any period of temporary acute changes in eGFR, whereas chronic slope intentionally excludes any acute effects.^54^^,^^55^ The eGFR 2-year chronic slope with sparsentan versus irbesartan reached statistical significance (–2.7 vs. –3.8 ml/min per 1.73 m^2^ per year, respectively; difference of 1.1 ml/min per 1.73 m^2^ per year; 95% CI, 0.1–2.1 ml/min per 1.73 m^2^ per year; *P* = 0.037).^8^ The difference in total eGFR slope did not reach statistical significance (–2.9 vs. –3.9 ml/min per 1.73 m^2^ per year, respectively; difference of 1.0 ml/min per 1.73 m^2^ per year; 95% CI, –0.03 to 1.94 ml/min per 1.73 m^2^ per year; *P* = 0.058), but was of similar magnitude to the chronic slope.^8^ An initial reduction in eGFR was seen in both the sparsentan and irbesartan arms, indicating that both treatments have an acute negative effect on eGFR. This may be due to their shared angiotensin II antagonism, and differences between the 2 agents in the attenuation of their acute negative effect over time could explain why statistical significance was achieved for the chronic but not the total eGFR slope.^8^^,^^55^^,^^56^

In the open-label extension study (NCT03762850),^36^ patients who completed the PROTECT trial were assessed for the long-term effects of sparsentan treatment (target dose of 400 mg/d) for up to 156 weeks (Kooienga L, Malecki R, Preciado P, et al. Concomitant sparsentan [SPAR] and sodium-glucose cotransporter-2 inhibitors [SGLT2is] in patients with IgA nephropathy [IgAN] in the PROTECT open-label extension [OLE] [poster]. Presented at: American Society of Nephrology Kidney Week. October 23–27, 2024; San Diego, CA. Poster FR-PO851).^8^ Recent results from a subgroup analysis of patients (n = 61) within this study who received sparsentan and concomitant SGLT2i treatment demonstrated greater reduction in proteinuria over 48 weeks.

#### Safety outcomes

TEAEs were reported in 187 (93%) of patients in the sparsentan group and 177 (88%) of patients in the irbesartan group.^8^ The most frequently (≥5%) reported events in patients receiving sparsentan compared with irbesartan included dizziness (30 [15%] vs. 13 [6%] patients) and hypotension (26 [13%] vs. 8 [4%] patients).^8^ TESAEs were reported to be similar between the 2 groups, occurring in 75 (37%) patients in the sparsentan group and 71 (35%) in the irbesartan group.^8^ Acute kidney injury occurred in 12 (6%) patients in the sparsentan group and 5 (2%) patients in the irbesartan group (4 [2%] vs. 1 [<1%] were serious, and 3 [1%] vs. none led to treatment discontinuation).^8^ There were no new safety signals observed, and TEAEs of edema, liver injury, and hyperkalemia were balanced between the sparsentan and irbesartan groups. A lack of new signals for liver injury with sparsentan is important given regulatory concerns over potential liver safety with some ETA receptor antagonists.^30^ No patients discontinued treatment because of heart failure or edema. One patient in the irbesartan group died due to cardiorespiratory arrest that was considered to be unrelated to the study drug; no deaths were reported in the sparsentan group.^8^

#### Biomarker data

Currently, no clinical biomarker data from the PROTECT trial have been published on the effects of sparsentan. Preliminary data from a study using an animal model that mimics human IgAN suggested that sparsentan may target immune and inflammatory processes that lead to protection from mesangial hypercellularity.^57^ Interim results from SPARTAN (NCT04663204),^58^ an open-label, single-arm exploratory study in 12 newly diagnosed, treatment-naïve patients with IgAN, also report reductions in urinary biomarkers (B-cell activating factor [BAFF], soluble complement 5b-9 [sC5b-9], soluble cluster of differentiaton 163 [sCD163], growth differentiation factor 15, CXC chemokine ligand 16, interleukin-6, and monocyte chemoattractant protein-1) with sparsentan from baseline over 24 weeks (Cheung CK, Barratt WA, Dhaun N, et al. Effect of sparsentan [SPAR] on proteinuria and urinary inflammatory and profibrotic biomarkers by baseline proteinuria strata in SPARTAN study participants with IgAN [poster]. Presented at: American Society of Nephrology Kidney Week. November 5–9, 2025; Houston, TX. Poster FR-PO0809), although further studies are needed.

### Key considerations when using sparsentan

In the United States, the US Food and Drug Administration mandates a risk evaluation and mitigation strategy when sparsentan is prescribed, which requires all patients to have liver function tests (aminotransferases and total bilirubin) before initiating treatment, monthly for the first 12 months, and then every 3 months during treatment with sparsentan. Pregnancy testing is also mandated before initiating treatment, monthly during treatment, and for 1 month after discontinuing treatment. Additionally, effective contraception is required before, during, and 1 month after discontinuation of treatment. In the European Union, sparsentan is contraindicated during pregnancy, and women of childbearing potential must use effective contraception during and up to 1 month after treatment has stopped. Patients should also be monitored for signs of liver injury.^34^

## Iptacopan

Iptacopan is a complement factor B inhibitor that is designed to work following “hit 4” of the 4-hit pathway.^46^ Dysregulation of the complement system plays an important role in IgAN, whereby the formation and deposition of immune complexes in the glomerular mesangium may directly activate the complement system, particularly the alternative complement pathway, and contribute to kidney inflammation and glomerular injury.^46^^,^^47^ Factor B has been shown to catalyze the formation of C3 convertases, which amplifies the alternative complement pathway. Iptacopan has received accelerated approval in the United States for the reduction of proteinuria in adults with primary IgAN at risk of rapid disease progression, generally a UPCR ≥1.5 g/g (Table 1). This was based on the results of the prespecified interim analysis of the phase 3 APPLAUSE-IgAN trial.^15^

### APPLAUSE-IgAN phase 3 trial

#### Trial design

APPLAUSE-IgAN (NCT04578834)^38^ is a multicenter, randomized, double-blind, placebo-controlled study.^37^ The study consists of a screening visit, a run-in period of up to 3 months, and a 24-month treatment period (Figure 1c).^37^ The trial was designed to evaluate the effect of iptacopan on proteinuria reduction and eGFR decline versus placebo. Adult patients with biopsy-confirmed IgAN, UPCR ≥1 g/g, eGFR ≥30 ml/min per 1.73 m^2^, and receiving stable supportive care for ≥90 days before study treatment were randomized (1:1) to receive oral iptacopan, 200 mg (n = 222), or placebo (n = 221) twice daily plus supportive care.^37^ Patients were vaccinated against *Neisseria meningitidis* and *Streptococcus pneumoniae*, and vaccinations against *Haemophilus influenzae* type B were performed according to local availability and regulations.^37^^,^^44^ A prespecified interim analysis was conducted when approximately 250 patients from the main study population had completed the 9-month visit. The study continued until all patients completed the 24-month treatment period, after which eligible patients could be entered into an open-label extension study.^37^

#### Prespecified interim analysis efficacy outcomes

The primary objective of the interim analysis was to demonstrate superiority of iptacopan (n = 125) versus placebo (n = 125) in reducing proteinuria at 9 months by measuring UPCR from a 24-hour urine collection.^37^ Iptacopan significantly reduced mean UPCR, relative to baseline, with a 38% (95% CI, 26%–49%; 2-sided *P* < 0.001) reduction seen versus placebo at month 9.^44^ Consistent with this analysis, the net reduction in UPCR in the first morning void from baseline at month 9 was 36% (95% CI, 23%–47%) with iptacopan versus placebo.^44^ At the time of publication, a press release from the manufacturer reported that iptacopan had demonstrated a “statistically significant, clinically meaningful improvement” in eGFR slope versus placebo in the 2-year APPLAUSE-IgAN trial.^59^ Full eGFR data are awaited.

#### Prespecified interim analysis safety outcomes

Safety was assessed in all the patients in the main trial population who had received at least 1 dose of iptacopan (n = 222) or placebo (n = 221) at the time of the data cutoff for the interim analysis. Iptacopan was well tolerated and had a favorable safety profile.^44^ TEAEs were reported in 138 (62%) and 153 (69%) of patients receiving iptacopan and placebo, respectively.^44^ These were mostly mild to moderate in severity, and no deaths were reported in either treatment arm. The most frequently reported TEAEs in patients receiving iptacopan versus placebo were coronavirus disease 2019 (COVID-19) (31 [14%] vs. 37 [17%]), upper respiratory tract infection (20 [9%] vs. 16 [7%]), nasopharyngitis (11 [5%] vs. 16 [7%]), headache (9 [4%] vs. 12 [5%]), and hypertension (4 [2%] vs. 13 [6%]), respectively.^44^ Few (<0.5%) patients had infections with microbiologic confirmation of encapsulated bacteria, and all of them recovered with antibiotics.^44^

#### Biomarker data

Phase 2 and 3 data indicated expected and sizable reductions in complement biomarker levels, including plasma Bb, serum Wieslab, and plasma and urinary C5b-9 or membrane attack complex, with iptacopan.^44^^,^^46^ As with other agents, studies of the potential association between their effects on biomarkers and clinical outcomes are needed.

### Key considerations when using iptacopan

In the United States, iptacopan is only available through a risk evaluation and mitigation strategy. Because of a potential increase in the risk of serious and life-threatening infections caused by encapsulated bacteria when receiving iptacopan, vaccinations for encapsulated bacteria (especially *N meningitidis* and *S pneumoniae*) need to be kept up to date before initiating treatment, and patients need to be monitored for early signs and symptoms of serious infections.

## Atrasentan

Atrasentan is a selective ETA receptor antagonist designed to reduce proteinuria by exerting antiproliferative, antifibrotic, and anti-inflammatory effects in patients with IgAN at risk of rapid disease progression.^16^^,^^39^ ETA receptor pathway activity appears to be elevated in the glomeruli and tubulointerstitial compartment in patients with IgAN and is associated with poor clinical outcomes, decreased eGFR, and increased proteinuria.^60^ The activation of ETA receptors by endothelin-1 has many pathologic downstream effects in IgAN, including immune cell migration, cytokine production, vascular smooth cell contraction, and mesangial cell activation.^60^ Atrasentan has received accelerated approval in the United States for the treatment of adult patients with IgAN at risk of rapid disease progression, generally a UPCR ≥1.5 g/g (Table 1). This is based on the results of the prespecified interim analysis of the ALIGN trial.^39^

### ALIGN phase 3 trial

#### Trial design

ALIGN (NCT04573478)^40^ is an ongoing multinational, double-blind, randomized, placebo-controlled study.^39^ The study consists of a screening period preceded by stratification of patients into a main stratum who received 12 weeks of stable, maximally tolerated RAS inhibition, and an exploratory stratum in which patients additionally received 12 weeks of a SGLT2i of the investigator’s choice.^39^ A period of 132 weeks on study drug with a 4-week washout phase followed randomization (Figure 1d).^39^ The trial aimed to determine the efficacy and safety of atrasentan compared with placebo in reducing proteinuria and preserving eGFR in patients with IgAN.^39^ A total of 340 patients aged ≥18 years with biopsy-proven primary IgAN, a total urine protein excretion of ≥1 g/d, and eGFR ≥30 ml/min per 1.73 m^2^ were included in the main stratum and were randomized (1:1) to receive atrasentan, 0.75 mg (n = 169), or placebo (n = 170) once daily, plus the maximum tolerated dose of RAS inhibitor.^39^ A prespecified interim analysis to investigate the primary end point of change in 24-hour UPCR from baseline to week 36 was conducted when 270 patients had completed the week 36 assessment or discontinued the study.^39^^,^^61^ A 48-week open-label extension study will be conducted after the end of the trial to allow for longer-term safety and efficacy monitoring.^39^

#### Prespecified interim analysis efficacy outcomes

Atrasentan (n = 135) demonstrated a clinically meaningful difference of –36% (95% CI, –45% to –26%) versus placebo (n = 135) in 24-hour UPCR change from baseline to week 36 (*P* < 0.001).^39^ The secondary eGFR and composite outcome end points will be assessed after the final study visit at week 136.^39^

#### Prespecified interim analysis safety outcomes

Safety outcomes were assessed in the entire main stratum (atrasentan, n = 169; placebo, n = 170), and atrasentan was associated with a favorable safety profile.^39^ AEs occurred in 139 (82%) of the atrasentan group and 144 (85%) of the placebo group, with 6 patients in each study group discontinuing treatment following an AE.^39^ Heart failure, fluid retention, and cardiac AEs are of special interest with ETA receptor antagonists.^39^ In ALIGN, no cases of heart failure were reported, and peripheral edema was balanced between the groups: 15 (9%) and 11 (7%) patients in the atrasentan and placebo groups, respectively.^39^ The most common reported AEs with atrasentan versus placebo were: COVID-19 (35 [21%] vs. 37 [22%]), nasopharyngitis (17 [10%] vs. 10 [6%]), peripheral edema (15 [9%] vs. 11 [7%]), anemia (11 [7%] vs. 2 [1%]), pyrexia (11 [7%] vs. 7 [4%]), and upper respiratory tract infection (11 [7%] vs. 9 [5%]).^39^

### Key considerations when using atrasentan

On the basis of data from animal studies, atrasentan may cause fetal harm, including birth defects and death; therefore, it is contraindicated during pregnancy.^32^ Before initiating atrasentan, pregnancy must be excluded and effective contraception should be used by all patients who can become pregnant before initiation of treatment, during treatment, and for 2 weeks after discontinuation of treatment with atrasentan.^32^

## Sibeprenlimab

Sibeprenlimab is a humanized IgG2 monoclonal antibody that selectively inhibits A proliferation binding ligand (APRIL) to inhibit B-cell signaling, which reduces circulating Gd-IgA1 in patients with IgAN.^41^ APRIL is a cytokine that promotes B-cell survival and differentiation.^41^^,^^62^ In patients with IgAN, blocking APRIL inhibits the formation of Gd-IgA1–containing immune complexes and mesangial deposition, leading to downstream inflammatory responses (including complement activation) that cause kidney injury, proteinuria, and loss of kidney function.^17^^,^^41^^,^^62^ In the United States, sibeprenlimab received accelerated US Food and Drug Administration approval in November 2025 to reduce proteinuria in adult patients with primary IgAN at risk of disease progression (Table 1).^17^^,^^63^ This approval was based on prespecified interim analysis of the VISIONARY trial.^63^ Sibeprenlimab is given as a 400-mg s.c. injection once every 4 weeks, with no dose adjustment required on the basis of sex, age, weight, race, or the presence of mild to moderate renal impairment (eGFR, 30–89 ml/min per 1.73 m^2^).^17^

### VISIONARY phase 3 trial

#### Trial design

VISIONARY is an ongoing phase 3 multicenter, double-blind, randomized trial of sibeprenlimab versus placebo.^41^ Patients were randomized 1:1 to receive either s.c. injection of sibeprenlimab, 400 mg (n = 259), every 4 weeks or placebo (n = 251) up to 100 weeks (26 total doses) (Figure 1e).^41^ Patients were stratified by level of UPCR (≤2 or >2 g/d) and eGFR (30–44 or ≥45 ml/min per 1.73 m^2^) at screening and use of SGLT2is (yes or no) at randomization.^41^ A maximally tolerated stable dose of an angiotensin-converting enzyme inhibitor or angiotensin receptor blocker was required for at least 3 months before screening and was continued throughout the study.^41^ The study aimed to evaluate the safety and efficacy of sibeprenlimab.^41^ The primary end point, analyzed at the interim analysis, was 24-hour UPCR at 9 months compared with baseline.^41^ The interim analysis reports on the first 320 patients from the main cohort.^41^ The key secondary end point of eGFR slope over 24 months has not been reported at the time of publication.^41^ Patients who completed VISIONARY (or the earlier phase 2 trial) can also continue sibeprenlimab treatment as part of a long-term, open-label extension study (NCT05248659^64^).^41^

#### Prespecified interim analysis efficacy outcomes

Sibeprenlimab (n = 152) demonstrated a 50% (95% CI, 44%–56%) reduction in 24-hour UPCR from baseline to month 9 (week 40) compared with a 2% (95% CI, –9% to 13%) reduction with placebo (n = 168).^41^ This corresponded to a 51% (97% CI, 43%–58%) lower adjusted geometric least-squares mean 24-hour UPCR with sibeprenlimab than placebo (*P* < 0.001).^41^ These results were consistent across the prespecified subgroups of screening 24-hour UPCR and eGFR and SGLT2i use.^41^

#### Prespecified interim analysis safety outcomes

Safety outcomes were analyzed in the main study population (n = 510), with the incidence of AEs similar between the 2 groups (74% and 82% in the sibeprenlimab and placebo groups, respectively).^41^ The AEs were mild to moderate in severity, with 1 (<1%) in the sibeprenlimab group and 4 (2%) in the placebo group leading to discontinuation.^41^ The most common (occurring in ≥10% in either group) AEs in the sibeprenlimab versus placebo group were upper respiratory tract infection (38 [15%] vs. 35 [14%]), injection-site erythema (34 [13%] vs. 30 [12%]), nasopharyngitis (32 [12%] vs. 25 [10%]), and injection-site pain (26 [10%] vs. 23 [9%]).^41^ A total of 25 (10%) patients in the sibeprenlimab group also had COVID-19 compared with 17 (7%) in the placebo group.^41^ In the VISIONARY trial, 88 of 256 (34%) evaluable patients receiving sibeprenlimab developed antidrug antibodies; of these, 21 patients (24%) developed antidrug antibodies that had neutralizing activity. Population pharmacokinetic analysis indicated that patients who developed antidrug antibodies had approximately 40% lower sibeprenlimab exposure than those who did not.^65^

#### Biomarker data

With sibeprenlimab, levels of serum Gd-IgA1, APRIL, IgA, IgG, and IgM all decreased relative to baseline from week 4 onward.^41^ At week 48, Gd-IgA1 was reduced by 67% (95% CI, 63%–71%), IgA by 69% (95% CI, 67%–71%), IgG by 35% (95% CI, 33%–37%), and IgM levels by 75% (95% CI, 73%–76%). APRIL suppression of >90% was seen with sibeprenlimab from week 4 and was maintained throughout treatment.^41^ By contrast, minimal change was seen in placebo patients.^41^

### Key considerations when using sibeprenlimab

Sibeprenlimab suppresses the production of Igs, thereby inhibiting the immune system, which may increase the risk of infections.^17^^,^^41^ Therefore, patients should be assessed for active infections before initiating this treatment.^17^ Additionally, sibeprenlimab may interfere with the effectiveness of vaccinations and increase vaccine-related infection risk. Live vaccines are not recommended to be given within 30 days of starting sibeprenlimab or during treatment.^17^ In the VISIONARY trial, despite both IgM and IgA levels being reduced by approximately 70% and IgG being reduced by approximately 35%, the rates of infection were similar between the sibeprenlimab and placebo groups.^41^

## Discussion

Following the recent identification and recognition by regulatory authorities of surrogate end points, eGFR slope and reduction in proteinuria are now being used to evaluate potential therapeutic candidates for accelerated approval for the treatment of IgAN. New therapies that have been specifically studied in IgAN across global populations have recently been approved following the use of these surrogate end points, whereas other potential agents are being evaluated in clinical trials. These therapies not only provide patients with a broader range of treatments but also notably allow clinical management of IgAN to target specific drivers of disease and nephron loss—in particular, targets within the multistep 4 “hits” process—which previously was not possible.

Nefecon, sparsentan, iptacopan, atrasentan, and sibeprenlimab are currently approved therapies for the treatment of IgAN (Table 1), based on phase 3 evidence demonstrating their efficacy and safety (Nawaz N, Thomas RC, Barratt J. Impact of nefecon on complement pathways in IgA nephropathy: an analysis of lectin, alternative, and terminal pathways [abstract]. Presented at: American Society of Nephrology Kidney Week. October 23–27, 2024; San Diego, CA. Abstract FR-PO859; and Thomas RC, Nawaz N, Barratt J. Specificity of nefecon in targeting pathogenic IgA in IgA nephropathy while preserving systemic humoral immunity [abstract]. Presented at: American Society of Nephrology Kidney Week. October 23–27, 2024; San Diego, CA. Abstract FR-PO894).^8^^,^^10^, ^11^, ^12^, ^13^, ^14^, ^15^, ^16^^,^^18^, ^19^, ^20^^,^^22^, ^23^, ^24^, ^25^, ^26^, ^27^, ^28^ Updated KDIGO 2025 guidelines recognize the beneficial effects of Nefecon and suggest a 9-month course of treatment for patients with IgAN who are at risk of progressive kidney function loss, to reduce the production of pathogenic forms of IgA and IgA immune complex formation and thus manage IgAN-specific drivers of nephron loss.^7^ Sparsentan has also shown beneficial effects on the generic response to IgAN-induced nephron loss by blocking both the endothelin and renin-angiotensin systems, resulting in a reduction in proteinuria and eGFR decline.^8^ The updated guidelines propose a simultaneous approach to managing patients with IgAN by targeting both specific drivers of nephron loss *and* generic risk of progressive kidney function loss.^7^ The recent KDIGO updated guidelines do not include iptacopan, atrasentan, or sibeprenlimab treatment for the management of IgAN because phase 3 data were not available when these treatments were being evaluated, but these should also be considered until recommendations for their use are included in subsequent guideline updates.

In addition to the advances made in increasing the number of approved and potential therapies for IgAN, it remains critical to identify biomarkers that correlate with clinical outcomes and are simple to use in clinical settings, as they could prove valuable in the management of patients with IgAN. This topic is discussed in more detail in the article, “The expanding role of biomarkers in the management of IgA nephropathy” by Jain and Rizk.^52^

## Disclosure

This article is published as part of a supplement sponsored by Calliditas Therapeutics, an Asahi Kasei company.

SN reports institutional grants from the IgAN Foundation, the American Society of Nephrology, and GlomCon Foundation; consulting fees from Alexion, Calliditas Therapeutics, Novartis, Otsuka, Sanofi, and Travere Therapeutics; speaker fees from Boehringer Ingelheim, Calliditas Therapeutics, Novartis, Otsuka, Sanofi, and Travere Therapeutics; and travel support from Calliditas Therapeutics and Otsuka. RAL reports institutional grants from Alexion, Amgen, Biogen, Calliditas Therapeutics, Novartis, Omeros, Otsuka, Roche, Travere Therapeutics, Vera Therapeutics, and Visterra; consulting fees from Alexion, Beigene, Biogen, Calliditas Therapeutics, Novartis, Omeros, Otsuka, Travere Therapeutics, Vera Therapeutics, and Vertex; honoraria from Calliditas Therapeutics, Novartis, Vera Therapeutics, and Vertex; travel support from Novartis, Takeda, and Vera Therapeutics; and serving on advisory boards for Amgen and Cara Therapeutics.
